# Bioactive Peptide VHVV Upregulates the Long-Term Memory-Related Biomarkers in Adult Spontaneously Hypertensive Rats

**DOI:** 10.3390/ijms20123069

**Published:** 2019-06-23

**Authors:** Da-Tong Ju, Ashok Kumar K., Wei-Wen Kuo, Tsung-Jung Ho, Ruey-Lin Chang, Wan-Teng Lin, Cecilia Hsuan Day, V. Vijaya Padma Viswanadha, Po-Hsiang Liao, Chih-Yang Huang

**Affiliations:** 1 Department of Neurological Surgery, Tri-Service General Hospital, National Defense Medical Center, Taipei 114, Taiwan; wxyz670628@yahoo.com.tw; 2 Graduate Institute of Basic Medical Science, China Medical University, Taichung 404, Taiwan; ashokbio93@gmail.com (A.K.K.); robert750927@hotmail.com (P.-H.L.); 3 Department of Biological Science and Technology, China Medical University, Taichung 404, Taiwan; wwkuo@mail.cmu.edu.tw; 4 Department of Chinese Medicine, Hualien Tzu Chi Hospital, Buddhist Tzu Chi Medical Foundation, Tzu Chi University, Hualien 970, Taiwan; tjho@mail.cmu.edu.tw; 5 School of Post-Baccalaureate Chinese Medicine, College of Chinese Medicine, China Medical University, Taichung 404, Taiwan; rueylin0112@gmail.com; 6 Department of Hospitality Management, College of Agriculture, Tunghai University, Taichung 407, Taiwan; 040770@thu.edu.tw; 7 Department of Nursing, MeiHo University, Pingtung 912, Taiwan; x00003023@mail.meiho.edu.tw; 8 Department of Biotechnology, Bharathiar University, Coimbatore 641 046, India; padma.vijaya@gmail.com; 9 Holistic Education Center, Tzu Chi University of Science and Technology, Hualien 970, Taiwan; 10 Cardiovascular research center, Hualien Tzu Chi Hospital, Buddhist Tzu Chi Medical Foundation, Hualien 970, Taiwan; 11 Department of Medical Research, China Medical University Hospital, China Medical University, Taichung 404, Taiwan; 12 Department of Biotechnology, Asia University, Taichung 413, Taiwan

**Keywords:** angiotensin-converting-enzyme inhibitor, bioactive peptide, blood–brain barrier, long-term memory

## Abstract

Hypertension is one of the growing risk factors for the progression of long-term memory loss. Hypertension-mediated memory loss and treatment remain not thoroughly elucidated to date. Plant-based natural compounds are an alternative solution to treating human diseases without side effects associated with commercial drugs. This study reveals that bioactive peptides extracted from soy hydrolysates mimic hypertension-mediated memory loss and neuronal degeneration and alters the memory molecular pathway in spontaneously hypertensive rats (SHR). The SHR animal model was treated with bioactive peptide VHVV (10 mg/kg/oral administration) and angiotensin-converting-enzyme (ACE) inhibitors (5 mg/kg/oral administration) for 24 weeks. We evaluated molecular level expression of brain-derived neurotrophic factor (BDNF), cAMP response element binding protein (CREB), and survival markers phospho-protein kinase B (P-AKT) and phosphoinositide 3-kinase (PI3K) after 24 weeks of treatment for SHR in this study. Western blotting, hematoxylin and eosin (H&E) staining, and immunohistochemistry showed long-term memory loss and neuronal degeneration in SHR animals. Bioactive peptide VHVV-treated animals upregulated the expression of long-term memory-relate proteins and neuronal survival. Spontaneously hypertensive rats treated with oral administration of bioactive peptide VHVV had activated CREB-mediated downstream proteins which may reduce hypertension-mediated long-term memory loss and maintain neuronal survival.

## 1. Introduction

Hypertension-mediated health problems are a major problem in the Western world. Hypertension causes a series of diseases in the brain, heart, and kidneys. Achieving a better understanding of hypertension-mediated diseases raises the possibility of therapeutic options [1]. Hypertension is a major risk factor for cerebrovascular disease and linked to neurodegenerative disease. Some other studies revealed that there was significantly more hypertension-mediated memory impairment in aged and diabetic conditions [2]. In normal people who only have hypertension, it alters brain-derived neurotrophic factor (BDNF)-tropomyosin receptor kinase B (TrkB) signaling in the hippocampus through structural and functional changes [3]. The blood vessels are more similar in the brain than the heart because of the greater change in disease severity in the brain. The cerebral arteries and arterioles are innervated with nerve sensory ganglia when hypertension alters blood pressure and arteries, and it can affect neuronal signaling in brain neurons [4]. 

Angiotensin-converting enzyme (ACE) inhibitors are globally accepted antihypertensive drugs used to treat coronary heart, renal, and vascular disease; some of the reports revealed another side of ACE inhibitors. It reverses arteriolar hypertrophy and promotes left ventricular hypertrophy in homo sapiens. Consecutive intake of ACE inhibitors could promote angioedema, pruritus, bullous eruptions, urticaria, photosensitivity, hair loss, and suppression of red blood cell production. Commercial ACE inhibitor use interrupts CNS signaling depending on the dose. Huntington's disease is one of the neuronal defects induced by the ACE inhibitor captopril. In recent years, various types of diseases are effectively treated with isolated herbal medicine, and there are potential herbal drugs used to treat memory loss, stroke, and gastrointestinal problems in humans [5]. It has been found that some of the isolated bioactive peptides in oyster and walnut can improve memory and oxidative stress [6,7,8]. Enzymatic hydrolysate for isolating bioactive peptides from soy protein extracts promotes cellular bioactive functions, such as antioxidant and cholesterol-lowering activities [7].

A lipolysis-stimulating peptide VHVV obtained from flavorzyme-soy protein (SPI) hydrolysate (F-SPIH) has potential lipolysis-stimulating and anti-obesity activities [9]. Although the soy-isolated peptide VHVV was digested by digestive enzymes pepsin and pancreatin, the lipolytic activity of VHVV is not affected by gastrointestinal enzymes [10]. The peptides can successfully cross the blood–brain barrier (BBB) and move from the blood to the brain; additionally, the bioactive peptide VHVV prevents and manages hypertension in an spontaneously hypertensive rats (SHR) animal model, in which the essential amino acids are greater than nonessential amino acids in the brain [11]. Essential amino acids are not synthesized in our body, and we need to obtain the bioactive peptide VHVV from the diet, as it is made of essential amino acids valine and histidine. The branched chain amino acid valine participates in many cellular processes, such as lipolysis, lipogenesis, glucose transportation, intestinal barrier glucose metabolism, function and absorption, mammary health, embryo development, and immunity [12,13]. Histidine improves hypoperfusion impairment in a mouse model; it regulates neurogenesis, astrocytes, and the integrity of the BBB [14]. Both of the amino acids are involved in many cellular processes, transport through the BBB, and regulate neuronal memory. Neuronal plasticity and long-time memory are basic processes in the brain that involve external sensory stimulation, socioemotional, and endocrine inputs from neuronal signals and mostly from a combination of all of these factors. Then, physicochemical processes alter relevant signaling pathways [15]. Long-term hypertension is the best model to understand long-term memory formation and learning. The formation of long-term memory (LTM) in humans depends on the formation of new synapses [15,16]. 

However, hypertension-regulated memory loss has not been fully studied. This study aimed to investigate the effects of bioactive peptide VHVV on long-term memory molecular pathway and neuronal cell survival in the SHR rat model. The SHR rats were treated with bioactive peptide VHVV for 24 weeks. The VHVV group showed significant expression of long-term memory biomarkers and survival responsible marker proteins. In SHR, all the survival and long-term memory proteins were expressed downstream. Enzymatic hydrolysis VHVV did not cause any defects in the treatment group. The administration of VHVV provides neuronal protection by regulating neuronal cell survival pathway. Our results indicate that VHVV enhance memory-related proteins in the rat cortex. The administration of bioactive peptide VHVV is considered to be a potential therapeutic agent to ameliorate hypertension-mediated long-term memory loss and neuronal death.

## 2. Results

### 2.1. Effect of VHVV and Hypertension-Mediated Defects in the SHR Rat Cortex

**Figure 1:**
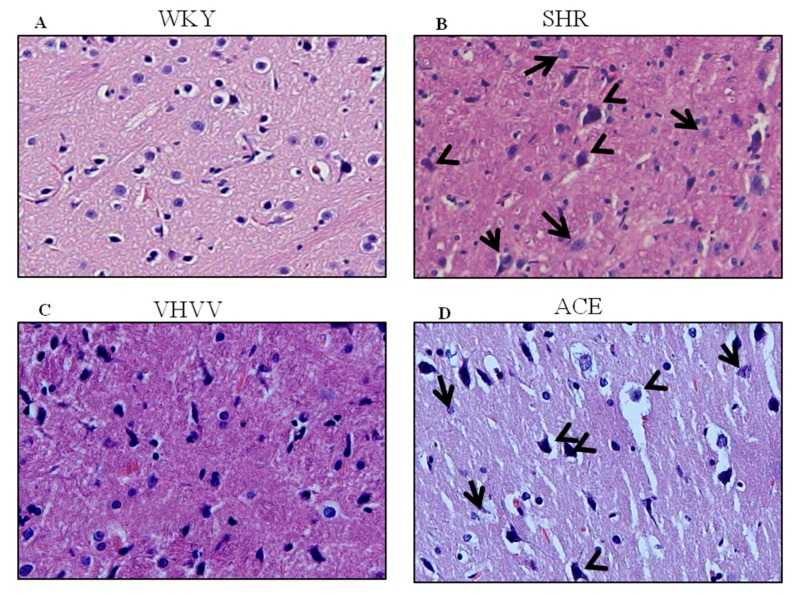
The hematoxylin and eosin (H&E) stain of the cerebral cortex (magnification: 400×): (**A**) The control Wistar Kyoto (WKY) group showed a good arrangement of neurons with clear round nucleus; (**B**) the spontaneously hypertensive rats (SHR) group showed neuronophagia, (short arrow), neurodegeneration (large arrow), and abnormal neuronal size (arrowhead; swollen). Of the neurons, (**C**) SHR rats treated with VHVV showed the rearranged neurons with better histopathological arrangement; (**D**) SHR rats treated with Angiotensin-converting enzyme (ACE) showed a moderate level of neurodegeneration (small arrow) and neuronal swollen (arrowhead).

To study the morphology of the brain, brain histopathology analysis was used in this study to confirm that the peptide VHVV can regulate morphological arrangements in the SHR rat model (Figure 1). SHR rats treated with biopeptide VHVV had better morphological rearrangement and interstitial space. Abnormal morphological arrangements such as neuronal swollen, neuronophagia, and neurodegeneration in SHR animals and ACE inhibitor-treated animals show a moderate level of neurodegeneration and neuronal swollen. This finding could represent hypertension-mediated neuronal degeneration and morphological changes in rat cortex.

### 2.2. Overexpression of BDNF Represents Long-Term Memory Biomarker in SHR through the Effect of VHVV

**Figure 2:**
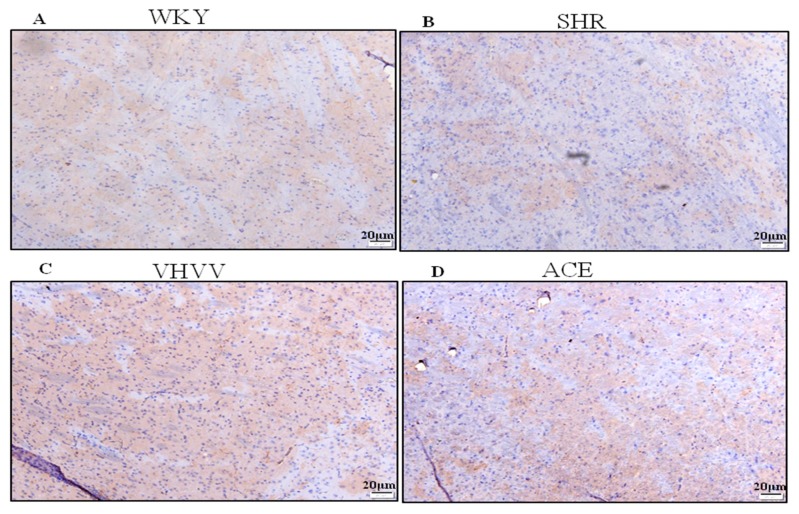
Immunohistochemistry (IHC) illustrating the expression level of brain-derived neurotrophic factor (BDNF) in the cortex. (**A**) The brown color indicated the expression and distribution of BDNF in tissue section. The scale bar is 20 μm. The expression of BDNF in the control WKY group. (**B**) SHR rat brain cortex shows visibly decreased BDNF levels compared to the WKY, VHVV, and ACE groups. (**C**) VHVV-treated rats with visibly enhanced BDNF compared to all other groups. (**D**) ACE-treated rats show more enhanced BDNF levels than the SHR group but visibly decreased levels compared to VHVV.

BDNF is important for neuronal structure maintenance and neuronal differentiation, and it is important for synaptic plasticity and long-term memory formation in the adult brain cortex. The result of immunohistrochimistry (IHC) showed a normal expression pattern of BDNF in the control group (Figure 2A). In the SHR group, the BDNF expression was reduced compared to control WKY and ACE inhibitor- and VHVV-treated animals (Figure 2B). The bioactive peptide VHVV-treated group showed an upregulated level of BDNF expression in tissue sections compared to all other treatment groups (Figure 2C). A moderate level of BDNF was shown in ACE inhibitor-treated animals (Figure 2D).

### 2.3. VHVV Regulates the CREB-Mediated Neuronal Cell Signaling Pathway in SHR Rats

**Figure 3:**
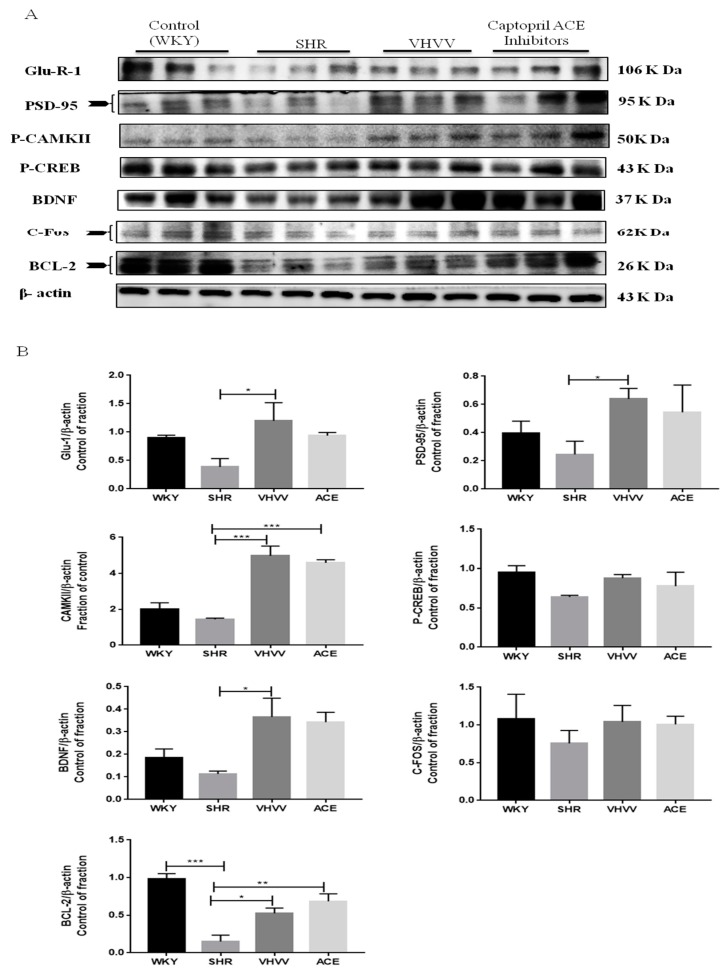
Protein expression levels of L-glutamate receptor 1 (Glu-R-1), postsynaptic density protein 95 (PSD-95), phosphor-Ca^2+^/calmodulin-dependent protein kinase II (P-CamKII), phosphor- cAMP response element binding protein (CREB)/ brain-derived neurotrophic factor (BDNF), C-Fos, and b-cell lymphoma-2 (BCL-2) and the corresponding Western blot analysis in the cortex of SHRs and WKY rats. (**A**) Long-term memory (LTM) progressively increased in bioactive peptide VHVV-treated animals compared with the positive control ACE inhibitor and control WKY groups. The 24-week SHR groups were significantly different from the VHVV- and ACE inhibitor-treated animals. (**B**) Quantification of Figure 3A. β-actin was used to monitor protein quantification as an internal control. The data are presented as the mean ± standard error. * *p* < 0.05, ** *p* < 0.01 and *** *p* < 0.001.

The bioactive peptide VHVV can regulate CREB-mediated upstream and downstream signaling molecules, such as CaMKII, PSD95, BDNF, C-Fos, BCL-2, and GluR-1 proteins, in the SHR rat model. Neuronal signaling transmitter, glutamine, is one of the important signaling transmitters which participate in the formation of long-term memory through regulating the CREB-mediated transcription process [17]. In Figure 3, the western blotting showed that all protein markers including CREB, CAMKII, PSD-95, BDNF, C-Fos, GluR-1, and BCL-2 were downregulated in SHR animals because the alteration of neuronal transmitters was affected by hypertension. The western blotting showed an equal level of downstream protein BDNF and C-Fos expression in the VHVV and ACE inhibitors groups and less BCL-2 the protein expression in the VHVV group compared to ACE inhibitors. The transcription factor CREB, the regulator proteins, CaMKII, transcription initiator PSD95, and GluR-1 showed a moderate level of protein expression in the VHVV-treated group compared to commercial drug ACE inhibitors and all other treatment groups. Furthermore, there was a significant difference between SHR-, VHVV-, and ACE inhibitor-treated animals; also, there was no significance difference between bioactive peptide VHVV and ACE inhibitors. Neuronal transmitters are potential regulators of long-term memory signaling pathways. All data were analyzed by one-way ANOVA, and the significant difference among the treatment was shown as * *p* < 0.05, * *p* < 0.01, and * *p* < 0.001.

### 2.4. TUNEL Compared with the Neuronal Survival Pathway Confirmed the Effect of Bioactive Peptide VHVV on the SHR Rat Cortex

**Figure 4:**
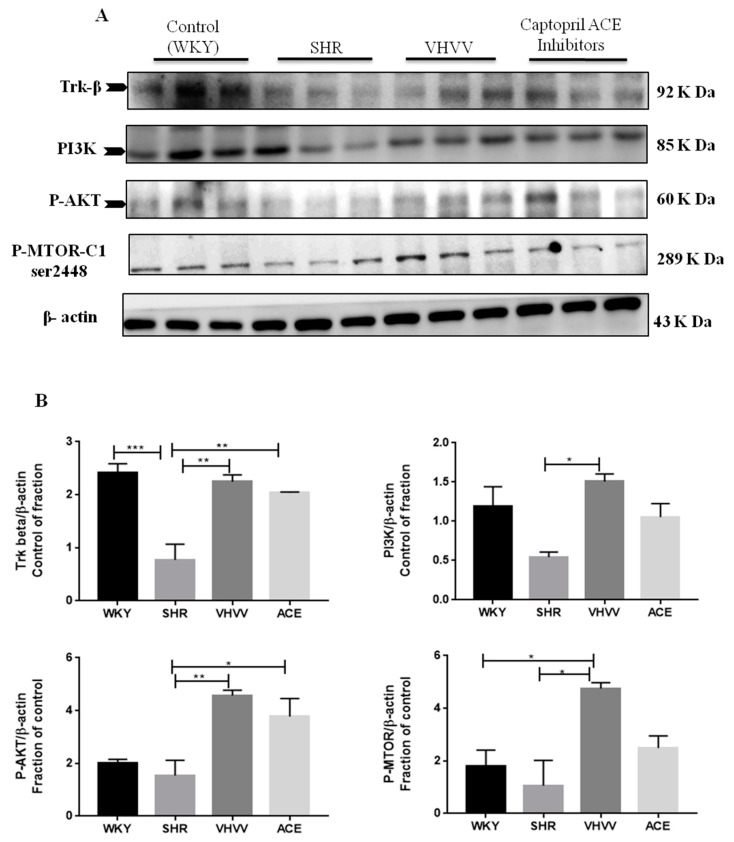
Effect of VHVV on neuronal cell survival through the PI3K-Akt-mTOR signaling pathway: Representative western blot results for tropomyosin receptor kinase B (Trk-β), phospho-protein kinase B (P-AKT), phosphoinositide 3-kinase (PI3K), and phospho-mechanistic target of rapamycin (p-mTOR). (**A**) Proteins are overexpressed in the VHVV group animals compared with all other groups and were significantly downregulated in the SHR groups compared with the positive control ACE and control WKY groups. (**B**) Western blot analysis of the relative optical density of protein expression was conducted by ImageJ software. The data are presented as the mean ± standard error. * *p* < 0.05; ** *p* < 0.01, and *** *p* < 0.001.

**Figure 5:**
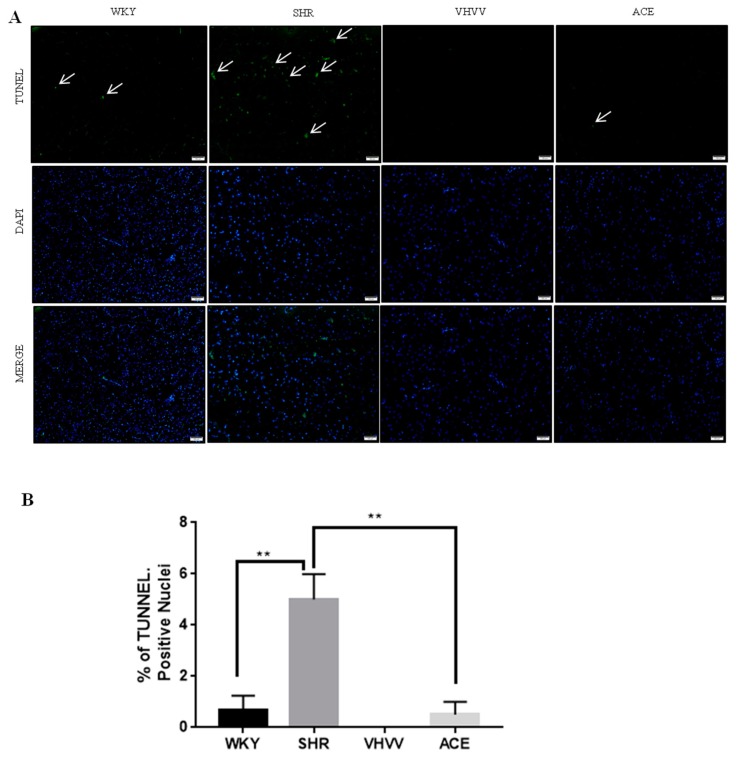
Photomicrographs of terminal deoxynucleotidyl transferase dUTP nick end labeling (TUNEL) staining in the experimental groups are shown. (**A**) Sections were labeled with TUNEL (green) to assess apoptotic brain cells. Sections were counterstained with DAPI (blue) to detect the nuclei. The arrowhead of SHR indicates TUNEL-positive cells; however, there is no TUNEL-positive cells in WKY and VHVV and a fewer number of positive cells in ACE. Scale bar showed as 100 μM. (**B**) Quantification of Figure 5A by image J software. ** *p* < 0.01.

To confirm that the Trk-β neuronal survival pathway was involved, receptor-mediated signaling markers PI3K, AKT, and M-TOR were found to activate CREB in neuronal cells. All marker proteins were expressed in the normal range in control WKY rats, and there were expected levels of expression in the bioactive peptide VHVV-treated animals. Downregulated levels of survival marker proteins quantified in SHR animals and the positive control ACE-inhibitor animals showed a high level of protein expression compared to SHR and control animals but was reduced compared to VHVV-treated animals (Figure 4). In tunnel assays, the DNA fragment gave green signals to indicate apoptosis and there were positive apoptotic signals in the tissue samples of the SHR animal, while the other groups showed tunnel-negative signals (Figure 5).

## 3. Discussion

In this present study, we hypothesized that bioactive peptide VHVV from soy hydrolysis could reverse hypertension-mediated long-term memory loss in molecular mechanism and neuronal cell death in rat cortex. A previous study showed the effect of the bioactive peptide VHVV on a young mouse model of fatty liver [18]. In the present study, we used SHR rats as a model system to study hypertension-mediated long-term memory loss. In 1998, Meneses and Hong reported that the SHR rats are a good model for studying hypertension-mediated learning and memory because the rats are bred using a conventional method in high-blood-pressure conditions [19]. Our results show that hypertension reduced the amount of BDNF, which is an important protein related with long-term memory in the cortex, and suggests that the hypertension impaired long-term memory, downregulated BDNF, and altered dendritic signals and neurogenesis in the cortex. In this study, the BDNF-Trkβ signaling proteins CaMKII, PSD95, BDNF, C-Fos, and BCL-2 and survival marker proteins PI3K, P-AKT, and p-mTOR were decreased in the SHR group (Figure 3 and Figure 4). Minichiello et al. reported before that BDNF-Trkβ signaling proteins are important for neuroplasticity and memory [20]. A previous report indicated the parietal cortex acts as a transient storage site for long-term information. However, the mechanisms of molecular memory formation have not been thoroughly elucidated to date [21]. The inferior temporal cortex of the brain increases structural and visual learning and mediates long-term memory through the expression of BDNF. However, motor learning and physical exercise also promote BDNF expression in the cortex and cerebellum [22]. In this study, we also found that the downstream protein of BDNF was expressed more in the bioactive peptide VHVV-treated animals, which was confirmed with immunohistochemical assay, and we concluded that our bioactive peptide VHVV potentially increased memory formation and neuronal cell survival by way of the overexpression of BDNF. Nevertheless, our data indicate that a 24-week treatment of bioactive peptide VHVV protects cortex neuronal cells against hypertension-mediated pathology. For example, the important transcription regulator CREB was expressed at a low level in the SHR group, but in the treatment group, it showed better expression compared to the control (Figure 2). Furthermore, the cortex region of VHVV-treated animals is resistant to hypertension-mediated defects. One of the important proteins, CaMKII, is abundant in the central nervous system and peripheral neurons and is one of the well-known documented molecules that regulate long-term memory (LTM) and memory [23]. Recent work reported that a CaMKII inhibitor reversed long-term memory and plays an important role in storage processes [24]. The function of CaMKII in the central nervous system remains mysterious, and indeed, Ca^2+^ stimulates CaMKII monomers and subsequently phosphorylates Thr286/Thr287 until the subunit dephosphorylates [25]. CaMKII regulates brain-derived neurotrophic factor (BDNF) by the phosphorylation of cAMP response element-binding protein (CREB); specifically, in vitro, CREB phosphorylates at Ser133 and Ser142 by the action of CaMKII, but in the case of neuronal culture, it phosphorylates CREB at Sers 133, 142, and 143 [26]. The phosphorylated CaMKII expression is greater in bioactive peptide VHVV animals, and it regulates transcription factor CREB in VHVV animals, which shows that the CREB-mediated downstream protein has greater BDNF expression in VHVV compared to the control and that SHR animals similar to the SHR show less expression compared to all treated animals (Figure 3). Furthermore, there is no significant difference between VHVV and the ACE inhibitor in all the marker proteins but there is a significant difference between SHR and VHVV. Glutamatergic- and GABAergic-mediated cellular mechanisms are important for cell survival and learning because they lead to increased survival and memory [27]. The cellular signaling-mediated neurogenesis was carried out by the activation of the Pi3K-AKT signaling pathway: The p-Akt (ser473) increased PI3K-mediated cell survival and protein synthesis and promotes plasticity and memory in a rat model [28]. Several studies reported that mTOR plays multiple roles in the brain, such as neuronal development, neuronal differentiation, synaptic plasticity, growth, and metabolism [29,30]. The mTORC1 pathway was activated by tyrosine kinase receptors (Trk) by induction of phosphoinositide-3-kinase (PI3K) and P-AKT [31]. In our study, the molecular proteins that are responsible for neuronal cell survival, such as PI3K-AKT-mTOR, were expressed more in the bioactive peptide VHVV-treated group compared to the SHR and ACE-treated animals. Among all of the treatment groups, the bioactive peptide VHVV had more effective expression than the commercial drug ACE inhibitors (Figure 4), which directly indicates that neuronal cells in SHR undergo neurodegeneration, as was confirmed by the terminal deoxynucleotidyl transferase dUTP nick end labeling (TUNEL) assay, in which fragmented nuclei were founded in SHR group animals (Figure 5). The results show that the PI3K-AKT-mTOR signaling pathway recovered neuronal tissue from hypertension-mediated cell death (Figure 4). In conclusion, hypertension is a well-known factor that induces brain abnormalities and cognitive impairment in old and middle-aged persons and alters structural and functional changes in the hippocampus by reducing BDNF-TrkB signaling [32]. In this study, we found an irregular histopathological structural arrangement in the SHR rat cortex due to the impairment in BDNF-TrkB signaling in the cortex by the effect of hypertension. According to the histopathological results, we hypothesized that hypertension not only affects memory formation but also alters the structural and functional changes in the rat cortex similar to how the bioactive peptide VHVV reverses hypertension-mediated neuronal degeneration and structural and functional changes (Figure 1). We found that both BDNF-Glutamate R-1 and TrkB-PI3K mediated signaling pathways are involved in long-term memory formation and cell survival through bioactive peptide VHVV. It is possible to use the bioactive peptide VHVV as a therapeutic drug used to treat hypertension-mediated long-term memory loss and neuronal degeneration. It is also suggested that the expression of BDNF in the cortex is a more effective way to approach hypertension-mediated defects. 

## 4. Materials and Methods

### 4.1. Materials

All chemicals, reagents, and materials were purchased from Sigma-Aldrich (St. Louis, MO, USA). The antibodies used in this study were GluR-1 (sc-13152 Santa Cruz, Texas, TX, USA), c-Fos (sc-52 Santa Cruz), Bcl-2 (610539), CREB-1 (sc-186 Santa Cruz), TrkB (ab134155, Cambridge, UK), p-mTOR-Ser2448 (#2971, Cell Signaling Beverly, Category, MA, USA), B-actin (sc-47778 Santa Cruz), BDNF (ab108319, Cambridge, UK), PI3K p85α (sc-423 Santa Cruz), P-AKT (#9275 Cell Signaling Beverly), PSD-95 (sc-32290 Santa Cruz), and CaM K II (05-532, Merck, Darmstadt, Germany). Secondary antibodies (Mouse, Rabbit, and Goat) were obtained from Invitrogen (Carlsbad, CA, USA).

### 4.2. Animal Experiments and Treatments

The male Wistar Kyoto rats (WKY) and spontaneously hypertensive rats (SHR) were procured from BioLASCO Taiwan Co. Limited Taipei, Taiwan. The animal was maintained at the China Medical University Animal Center in appropriate conditions. The bioactive peptide VHVV was purchased from DG peptides Co. Ltd., China. Prepared bioactive peptides were administered alternative days for 24 weeks. Animals were grouped in 3 different treatment groups: WKY (control), SHR (control), bioactive peptide-treated group VHVV, (10 mg/kg/alternative days), and a positive control treated with ACE inhibitors (5 mg/kg/alternate days). All animals were fed normal tap water and standard laboratory animal feed and kept in a 22–24 °C habitat temperature-maintained cage under 12-h light and dark cycles. After 24 weeks of treatment, animals were sacrificed later with terminal anesthesia. All animal experiment protocols were approved by the Institutional Animal Care and Use Laboratory Animals (National Institutes of Health Publication) committee of China Medical University, Taichung, Taiwan (No. 101-263-B).

### 4.3. Immunohistochemistry

After animal sacrifice, the brain was soaked in formalin after dehydration and 3-μm-thick microtome TMA slides were embedded in paraffin wax. The slides were individually immunostained with anti-ANP antibodies, employing the Ultra Vision LP Detection System (Vector Laboratories, Waltham, MA, USA). The embedded tissue was kept overnight at 60 °C for the pull-out embedded paraffin. After overnight rehydration with serious xylene and graded ethanol, “H_2_O_2_” blocking buffer was used to block peroxidation on embedded tissue and then washed with “ddH_2_O”. The slides were treated with citrate buffer by heating with a microwave for the required time interval after cooling at room temperature. These slides were incubated with primary antibody for 2 h (1:100) after blocking with blocking buffer. Thereafter, the slides were incubated with the universal secondary antibodies for 30 min before staining with hematoxylin, and the slides were stained with chromogen for 5 min. Finally, slides were counterstained with hematoxylin, dehydrated by a series of graded ethanol to xylene washes, and fixed on coverslips with mounting media (Sigma Chemical, Saint Louis, MO, USA). Mounted tissue sections were viewed under a microscope (magnification 200×).

### 4.4. Hematoxylin and Eosin Staining

The embedded brain tissue was deparaffinized followed by dehydration with a graded alcohol and xylene wash. The tissue sections were stained with the determination of the brain structure, and tissue sections were stained with hematoxylin–eosin. The tissue slides were dehydrated by an ethanol and xylene wash after gentle washing with water. Thereafter, the slides were mounted with a coverslip, and prepared samples were examined under a microscope. Tissue morphological changes were identified by using hematoxylin and eosin (H&E) staining.

### 4.5. Terminal Deoxynucleotidyl Transferase (TdT)-Mediated dUTP-Biotin Nick end Labeling

The embedded paraffin brain tissue section was used to detect apoptosis-positive signals using a TUNEL assay (in situ cell death detection kit, fluorescein, Roche, Mannheim, Germany) performed using the manufacturer’s protocol. The tissue was kept overnight at 60 °C to unwax and rehydrate according to kit protocol and was washed with xylene and different percentages (100, 95, 75, and 55%) of ethanol and double distilled water for 10 min. Mounted TUNEL tissue was contained with Proteinase K (ZYMED, Waltham, MA, USA) for 30 min at room temperature and then washed twice with PBS for 5 min each. Sodium citrate (0.1%, Sigma, Saint Louis, MO, USA) was treated on the tissue slide for 8 min, followed by washing twice for 5 min with PBS. After the PBS wash, slides were stained with a TUNEL reaction mixture (1:9/enzyme/reaction solution) at 37 °C and maintained in the dark for 1 h, followed by two 5-min washes with PBS. Slides were stained with 4’,6-diamidino-2-phenylindole (DAPI, Sigma) for 5 min and further washed as mentioned before. The slides were analyzed under a fluorescence microscope, and fragmented TUNEL-positive nuclei green signals were amplified in the 450–500 nm range.

### 4.6. Tissue Protein Extraction

Collected brain tissues were washed with phosphate-buffered saline solution (PBS). The cortical region was separated from whole brain tissue with proper guidance. Lysis buffer (0.002 M EDTA, 0.02 M Tris, 10% glycerol, 0.05 M 2-mercaptoethanol, protease inhibitor per 10 mL, and phosphatase inhibitor 1 μL per mL) was prepared based on a previous report, and a volume of prepared lysis buffer (100 mg/ mL) was added and homogenized. Each sample of lysis was collected and kept on ice for 30 min. The sample tubes were centrifuged at 12,000 rpm for 40 min, and the optimum temperature was maintained at 4 °C. Supernatants were separated from the pellet and stored at −80 °C for future experiments. The amount of protein was calculated to use the Lowry quantification assay with the standard protocol.

### 4.7. Lowry Protein Assay

Bovine serum albumin (BSA) solution (0.5 mg/mL) was prepared and mixed with the proper amount of water and 2 mg/mL of BSA. The standard curves were obtained based on ascending grades of protein standards (0, 0.1, 0.2, 0.3, 0.4, and 0.5 mg/mL) using diluted BSA solution at a concentration of 0.5 mg/ml. In the first step, alkaline copper tartrate solutions were prepared and added exact amount of Na_2_CO_3_ (2% *w*/*v*) in NaOH (0.1 M) with CuSO_4_, 5H_2_O (1% *w*/*v*) and then Na-K tartrate (1% *w*/*v*) at a ratio of 98:1:1. The 10-min RT tetradentate complex bound with the central atom of copper (Cu^2+^ and Cu^1+^) to form alkaline and copper tartrate complexes. Second, Felon's reagent was added 30 min after RT. After 30 min, Cu^1+^ ions were reduced in the solution with phosphomolybdic–phosphotungstic, and blue color formation was measured at absorbances of 405 to 750 nm.

### 4.8. Western Blot Analysis

The quantified protein was prepared (30 μg) and mixed with 5% SDS gel loading dye. The prepared mixture was placed on a 100 °C hot pot for 10 min. An equal amount of sample was loaded and separated via 6–12% sodium dodecyl sulfate-polyacrylamide gel electrophoresis (SDS-PAGE). The SDS page gel percentage change depends upon the protein of interest. The separated protein was electroblotted from gel to polyvinylidene difluoride (PVDF, GE Healthcare, Amersham, UK) after transfer blocking with 5% skim milk for 1 h at RT. The transfer membrane was kept overnight at 4°C with primary antibody (Antibody dilution, 1: 1000). Thereafter, the membrane was washed with PBS 3 × 10 (3 times for 10 min each) and conjugated with a secondary antibody for 1 h at RT. (Antibody dilution, 1:10,000). The PBS wash was repeated for the blot with Western Chemiluminescent HRP Substrate (Millipore, Burlington, MA, USA). Every membrane was restored with prepared a stripping buffer (Pierce, Rockford, IL, USA) with a standard protocol and processed with different antibodies for new results. The images were cropped by Adobe Premiere 7.1. The results were appraised using ImageJ software (NIH, Bethesda, State of Maryland, USA). 

### 4.9. Statistical Analysis

All statistical analyses were performed with three different values from different animals in each group. Multiple comparisons were done by one-way ANOVA with GraphPad Prism 5 statistical analysis software (San Diego, CA, USA). A *p*-value <0.05 was considered to be significant. 

## 5. Conclusions

In conclusion, based on the current study, bioactive peptide VHVV potentially prevents injury from hypertension by activating the long-term memory-related molecular pathway. Our results showed that treatment with VHVV upregulates the expression of BDNF, which is an important protein related with long-term memory and neuronal cell death in hypertension rat cortex. These findings hint that the bioactive peptide VHVV may enhance long-term memory and neurogenesis in hypertensive rats. We further hypothesize that bioactive peptide VHVV might be used as an alternative drug to commercial drug ACE inhibitors for preventing hypertension-mediated impairment.

## Abbreviations

ACE	angiotensin converting enzyme
AKT	protein kinase B
BBB	blood–brain barrier
Bcl-2	B-cell lymphoma 2
BDNF	brain-derived neurotrophic factor
CAMK	Ca^2+^/calmodulin-dependent protein kinase
CREB	cAMP response element-binding protein
DAPI	4′,6-diamidino-2-phenylindole
EDTA	ethylenediaminetetraacetic acid
GluR-1	glutamate receptor 1
LTM	long-term memory
mTOR	The mammalian target of rapamycin
PI3K	phosphoinositide 3-kinases
PVDF	polyvinylidene difluoride
PSD-95	postsynaptic density protein 95
SHR	spontaneously hypertensive rat
TrkB	tropomyosin receptor kinase B
WKY	wistar kyoto rats
